# “IT CAN BE A CHAMELEON”: LEWY BODY DEMENTIA PRESENTATION WITH AN ELUSIVE CLINICAL PROFILE—CASE REPORT AND DISCUSSION

**DOI:** 10.1093/geroni/igad104.2254

**Published:** 2023-12-21

**Authors:** Javier Malcolm Barney, Mario Moya, Jessica Martin, Andrew Dentino

**Affiliations:** DHR Health, Edinburg, Texas, United States; DHR Health, Edinburg, Texas, United States; Rio Grande Valley Graduate Medical Education Consortium, Edinburg, Texas, United States; Rio Grande Valley Graduate Medical Education Consortium, Edinburg, Texas, United States

## Abstract

Lewy body dementia is a progressive neurodegenerative disease and is considered to be the second most common cause of dementia in the population above 65 years of age. It remains a challenging and complicated disease process to profile clinically with an elusive diagnosis that shares similarities with Alzheimer’s, leading to patients being misdiagnosed. The decline is compounded by inappropriate coordinated care efforts, and therefore ill preparing the patient and negatively affecting their relatives and support groups. We describe the case of an 83-year-old Hispanic male who over the course of 32 months sustained multiple injuries due to falls landing him in the emergency department; receiving numerous consultations from hospitalists, surgeons, infectious disease specialists, neurologists, psychiatrists, and radiologists. A combined blend of inpatient and outpatient visits was not enough to circumvent the recurrent theme that plagues similar cases worldwide with non-reliable criteria that can identify Lewy Body Dementia early in the presentation of the disease. The first mention of DLB was almost at the 32 months mark into the patient’s journey at this particular Hospital System. A multidisciplinary approach with enhanced communications and round table discussions should be a quality of care improvement initiative to help better serve this population of patients. DLB’s rapid progression and sensitive drug therapy parameters should place this as equally scalable as other multi-specialty collaborations such as tumor board, limb salvage committee, ethics, and Trauma review.

